# Investigation of multi-trait associations using pathway-based analysis of GWAS summary statistics

**DOI:** 10.1186/s12864-018-5373-7

**Published:** 2019-02-04

**Authors:** Guangsheng Pei, Hua Sun, Yulin Dai, Xiaoming Liu, Zhongming Zhao, Peilin Jia

**Affiliations:** 1 Center for Precision Health, School of Biomedical Informatics, The University of Texas Health Science Center at Houston, 7000 Fannin St. Suite 820, Houston, TX 77030 USA; 2 Human Genetics Center, School of Public Health, The University of Texas Health Science Center at Houston, Houston, TX 77030 USA; 3 Department of Biomedical Informatics, Vanderbilt University Medical Center, Nashville, TN 37203 USA

**Keywords:** GWAS, Pathway enrichment analysis, Multi-dimensional scaling, Cross-trait association, Summary statistics, Pleiotropy abbreviations

## Abstract

**Background:**

Genome-wide association studies (GWAS) have been successful in identifying disease-associated genetic variants. Recently, an increasing number of GWAS summary statistics have been made available to the research community, providing extensive repositories for studies of human complex diseases. In particular, cross-trait associations at the genetic level can be beneficial from large-scale GWAS summary statistics by using genetic variants that are associated with multiple traits. However, direct assessment of cross-trait associations using susceptibility loci has been challenging due to the complex genetic architectures in most diseases, calling for advantageous methods that could integrate functional interpretation and imply biological mechanisms**.**

**Results:**

We developed an analytical framework for systematic integration of cross-trait associations. It incorporates two different approaches to detect enriched pathways and requires only summary statistics. We demonstrated the framework using 25 traits belonging to four phenotype groups. Our results revealed an average of 54 significantly associated pathways (ranged between 18 and 175) per trait. We further proved that pathway-based analysis provided increased power to estimate cross-trait associations compared to gene-level analysis. Based on Fisher’s Exact Test (FET), we identified a total of 24 (53) pairs of trait-trait association at adjusted *p*_FET_ < 1 × 10^− 3^ (*p*_FET_ < 0.01) among the 25 traits. Our trait-trait association network revealed not only many relationships among the traits within the same group but also novel relationships among traits from different groups, which warrants further investigation in future.

**Conclusions:**

Our study revealed that risk variants for 25 different traits aggregated in particular biological pathways and that these pathways were frequently shared among traits. Our results confirmed known mechanisms and also suggested several novel insights into the etiology of multi-traits.

**Electronic supplementary material:**

The online version of this article (10.1186/s12864-018-5373-7) contains supplementary material, which is available to authorized users.

## Background

Genetic variants that affect multiple traits are often called pleiotropy [1]. It has been increasingly recognized that pleiotropic effects are likely widespread in human complex traits [1, 2]. For example, individuals carrying schizophrenia risk alleles tended to be associated with increased risk of Crohn’s Disease and Ulcerative Colitis [3]; men with cystic fibrosis are often infertile because of congenital absence of the vas deferens [4]. The past decade has witnessed a wave of genome-wide association studies (GWAS), generating rich resources of genetic variants in large cohorts with various clinical phenotypes or traits. GWAS data have been successful in identifying disease-associated genetic variants, pinpointing biological mechanisms, explaining heritability [5], and further, they have greatly promoted the investigation of pleiotropy systematically. One of the most well-known loci is the Major Histocompatibility Complex (MHC) region on chromosome 6, which was identified to be associated with many traits through GWAS [6]. Recently, Pickrell et al. used large GWAS data to perform a systematic search for genetic variants that influence pairs of 42 traits and to interpret the associations [3]. Mancuso et al. introduced a method to estimate the local genetic correlation based on gene expression and utilized it to 30 traits [7]. We recently applied functional and regulatory approaches to identify novel regulatory variants in large-scale genome-phenome (GWAS-PheWAS) studies [8]. Notably, the cross-trait association is highly relevant, but not identical, to pleiotropy because trait association derived from shared genetic variants does not require the underlying causal mechanism. Yet pleiotropy occurs when a genetic variant causes multiple traits. In this study, we utilized GWAS data to estimate cross-trait association at the pathway level, aiming to reveal biological and functional insights towards the understanding of pleiotropic effects.

GWAS data provide systematic, unbiased measurement of genome-wide variants. However, the marginal effect of each single nucleotide polymorphism (SNP) is often small to moderate, making it challenge to study cross-trait associations at the SNP level. Furthermore, SNP level analyses are typically less informative for biological interpretation. For example, > 80% of disease-associated variants identified from GWAS are located in non-coding regions and for the few variants that are located in coding regions, the biological mechanisms often remain unclear [9, 10]. There is thus an urgent need for methods that could fill in the gap between GWAS discoveries and clinical applications, i.e., methods of precision medicine [11]. Grouping of multiple SNPs across genes or well-established pathways can be beneficial to improve statistical power and gain insight into a biological system [12–14]. Over the past few years, numerous pathway enrichment methods have emerged to greatly boost GWAS data discovery and better understanding of the molecular basis of phenotypes [15, 16], such as gene set enrichment analysis (GSEA) and ALIGATOR (Association LIst Go AnnoTatOR) [17]. These algorithms have greatly contributed to investigate the common associated pathways in different traits and diseases. For example, the Network and Pathway Analysis Subgroup of the Psychiatric Genomics Consortium (PGC) has employed five pathway enrichment methods to study the common pathways across multiple psychiatric disorders, providing novel insights into the etiology of psychiatric disorders [11]. However, methods to investigate the combined effect of multiple SNPs, either at the gene or the pathway levels, are universally subjected to influential factors such as gene length, pathway size, SNP density, and linkage disequilibrium (LD) [18, 19]. In addition, such strategies have not been extended to cross-trait associations systematically.

In this work, we collected GWAS summary statistics data for 25 traits and developed an analysis framework to investigate the cross-trait associations using two complementary pathway-based enrichment analysis methods. We utilized a comprehensive annotation of pathways while controlling for pathway redundancy. Our results reported molecular pathways that were implied by genetic variants underlying multiple traits and provided insights for the understanding of cross-trait association and future clinical applications.

## Results

### Analysis workflow

**Fig. 1:**
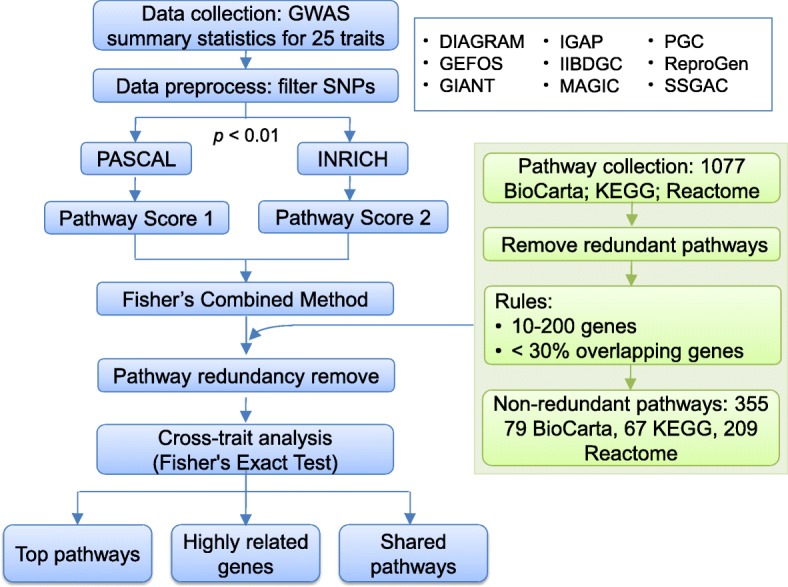
Overview of the analysis workflow. Details are provided in the Materials and methods section

Figure 1 illustrated our analysis framework. We started with the GWAS summary statistics for 25 traits downloaded from nine consortia. We grouped these traits into four general groups (Table 1): (i) six anthropometric and social traits: Body Mass Index (BMI), Bone Mineral Density in Female Neck (FN-BMD), Bone Mineral Density in Lumbar Spine (LS-BMD), Educational attainment (EDU), Height, and Waist–Hip Ratio (WHR); (ii) three immune-related traits: Crohn’s Disease (CD),, Rheumatoid Arthritis (RA), and Ulcerative Colitis (UC); (iii) 10 metabolic traits: Age At Menarche (AAM), Coronary Artery Disease (CAD), Fasting Glucose (FG), Fasting Insulin (FI), High-density Lipoproteins (HDL), Low-density Lipoproteins (LDL), Total Cholesterol (TC), Triglycerides (TG), Type 1 Diabetes (T1D), and Type 2 Diabetes (T2D); and (iv) six neurological/neuropsychiatric phenotypes: Alzheimer’s disease (ALZ), Attention Deficit-Hyperactivity Disorder (ADHD), Autism Spectrum Disorder (ASD), Bipolar Disorder (BD), Major Depressive Disorder (MDD), and schizophrenia (SCZ). We summarized features of these datasets in Table 1, including sample size, the number of SNPs available with statistical significance, and the publication year. All data were conducted using samples of European ancestry. The number of SNPs with summary statistics ranged from ~ 1 million to ~ 12 million per trait.

**Table 1:** Summary of the 25 traits

Phenotype	Abbreviation	# individuals (cases/controls, if applicable)	# Total SNPs	# Sig. SNPs^1^	Generation year
1 Neurological/Neuropsychiatric phenotypes					
Alzheimer’s disease	ALZ	17,008 / 37,154	7,055,881	108,745	2013
Attention deficit-hyperactivity disorder	ADHD	1947 trio / 1947 trio & 840 / 688	1,230,535	13,120	2013
Autism spectrum disorder	ASD	4788 / 4788	1,245,864	15,781	2013
Bipolar disorder	BD	6990 / 4820	1,233,533	18,723	2013
Major depressive disorder	MDD	9227 / 7383	1,232,793	14,864	2013
Schizophrenia	SCZ	9379 / 7736	1,237,958	24,361	2013
2 Anthropometric and social traits					
Body mass index	BMI	322,154	2,554,637	63,111	2015
Bone mineral density (femoral neck)	FN-BMD	32,735	10,586,899	117,114	2015
Bone mineral density (lumbar spine)	LS-BMD	28,498	10,582,866	120,390	2015
Educational attainment	EDU	293,723	8,146,840	311,622	2015
Height	HEIGHT	253,288	2,550,858	239,924	2014
Waist–hip ratio	WHR	142,762	2,560,788	38,995	2015
3 Immune-related traits					
Crohn’s disease	CD	5956 / 14,927	12,276,505	185,729	2015
Rheumatoid arthritis	RA	18,136 / 49,724	8,747,962	122,601	2014
Ulcerative colitis	UC	6968 / 20,464	12,255,196	181,092	2015
4 Metabolic phenotypes					
Age at menarche	AAM	182,416	2,441,815	44,202	2015
Coronary artery disease	CAD	22,233 / 64,762	2,420,360	41,279	2011
Fasting glucose	FG	46,186	2,470,476	32,818	2010
Fasting insulin	FI	38,238	2,461,105	30,044	2010
High-density lipoproteins	HDL	99,900	2,692,429	39,833	2010
Low-density lipoproteins	LDL	95,454	2,692,564	40,261	2010
Total cholesterol	TC	100,184	2,692,413	44,112	2010
Triglycerides	TG	96,598	2,692,560	40,574	2010
Type 1 diabetes	T1D	9934 / 16,956	2,048,237	45,430	2011
Type 2 diabetes	T2D	12,171 / 56,862	2,473,440	39,081	2012

^1^Significant SNPs (*p* < 0.01)

Our ultimate goal is to investigate the shared genetic components of multiple traits at the pathway level. To this end, we employed two methods to conduct the pathway-based analysis, PASCAL (PAthway SCoring ALgorithm) [12] and INRICH (INterval-based enRICHment) [20]. PASCAL evaluates pathways based on the combined effect of multiple variants and genes, which may individually represent weak associations with the investigated trait [12]. Conversely, INRICH considers genes by using the most significant SNP located within the gene and detects pathway enrichment based on genes with strong association [20]. We chose SNPs with *p*-value < 0.01 for the following analysis. A comprehensive collection of pathways was included, consisting of 1077 pathways from BioCarta, KEGG, and Reactome databases. To reduce the redundancy among pathways, we conducted pairwise comparison and chose those pathways with size within the range of 10–200 genes and sharing no more than 30% genes with other pathways. This step resulted in 355 pathways considered as nearly non-redundant, including 79 BioCarta, 67 KEGG, and 209 Reactome pathways. Pathways that were identified as significantly associated with a trait by both methods were considered as with robust evidence. We calculated a combined *p*-value for each pathway using Fisher’s combined probability test [21]. With these combined *p*-values per pathway per trait, we then applied Fisher’s Exact Test (FET) to estimate whether any two traits shared a significant number of pathways that were significantly associated with each of them. We used Benjamini and Hochberg’s method [22] to correct multiple tests.

### Pathway enrichment analysis

**Fig. 2:**
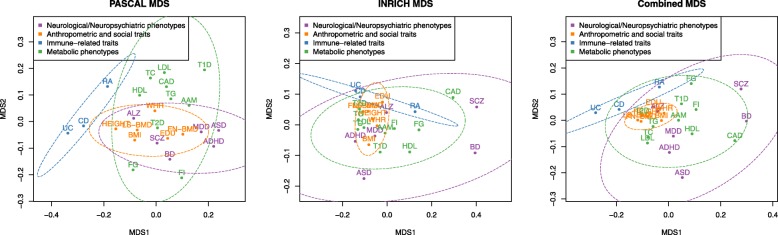
Multi-dimensional scaling (MDS) plot for all 25 traits using the results of PASCAL, INRICH, and the combined method. The *x*-axis and *y*-axis represent the first and second dimension from MDS results, respectively. The ellipse indicates the confidence limit standard deviations at 0.95 multiplied with the corresponding value found from the chi-squared distribution with 2 degrees of freedom

To explore the pathways that were respectively identified as significantly associated with each trait by PASCAL and INRICH, we conducted a multi-dimensional scaling (MDS) analysis [23]. As shown in Fig. 2, traits from the same group in general were located closer to each other than to traits from different groups. For example, immune-related traits CD and UC as the most common forms of inflammatory bowel disease (IBD) and RA formed a unique cluster that was quite distinct from other trait groups based on both the PASCAL results and the INRICH results, as well as the combined method. The other three groups did not show clear boundaries in location but a trend of cluster was observed. For example, in the PASCAL results, 7 out of 10 metabolic traits were distinguishable from other groups, while T2D, FI, and FG were scattered in other areas. In the combined results, the six anthropometric and social traits formed a concise cluster that can be visually identified, although their locations in the MDS plot overlapped with the metabolic traits and neurological/neuropsychiatric traits. The six neurological/neuropsychiatric phenotypes were not closely located in all three result sets, but sub-clusters were observed. For example, SCZ and BD were relatively closer to each other in all cases, which is consistent with previous reports that shared genetic liabilities exist between SCZ and BD [24]. ALZ appeared as an outlier located far away from any other neuropsychiatric traits. This is expected because, although all six traits are neurological/neuropsychiatric related, ALZ is the only neurodegenerative disease that involves the degeneration of multiple regions in brain. Thus, the pathways involved in ALZ are likely different from those involved in ADHD, ASD, BP, MDD, and SCZ.

Although PASCAL and INRICH differ in the selection of gene signals and the association tests, the results from the two methods are overall consistent and the main patterns were reproducible. The number of trait-associated pathways identified by PASCAL and INRICH showed a *spearman* correlation of 0.621 (*p*-value = 7 × 10^− 4^), indicating the robustness of our results. For example, the immune group tended to have more pathways than other disease groups did and this trend was detected by both methods.

### The most significant pathways associated with traits

**Fig. 3:**
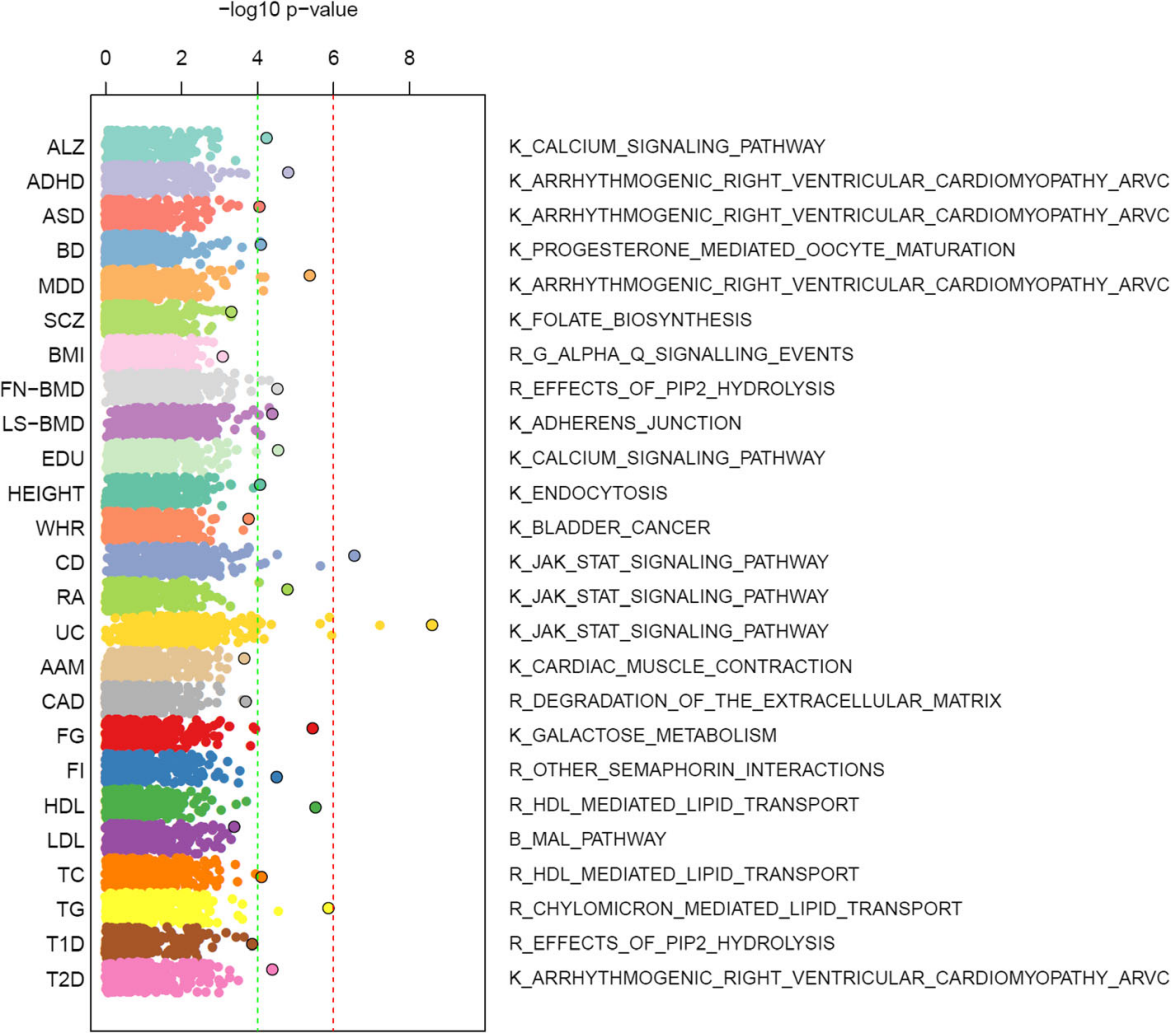
Distribution of pathway-based analysis results in each trait. The *x*-axis represents the -log_10_ transformed *p*_combined_ for pathways. The most significantly associated pathway with each trait was labeled. The first letter in each pathway name indicates the sources (K for KEGG, R for Reactome, and B for BioCarta, respectively)

To further explore what pathways were associated with each trait, we identified the most significantly associated pathway in each trait. Figure 3 summarizes the whole distribution of all pathways by traits. The detailed results were provided in Additional file 1: Table S1. At *p*_combined_ *<* 0.01, we found an average of 54 pathways per trait (ranged between 18 and 175). Among them, only HDL and BD had < 25 disease-associated pathways. LS-BMD, CD and UC each had more than 100 disease-associated pathways. Interestingly, the most significant pathways of traits that belong to the same category tended to be the same or similar. For example, in neurological/neuropsychiatric traits, the KEGG pathway “arrhythmogenic right ventricular cardiomyopathy (ARVC)” was the most significantly associated with ADHD (*p*_combined_ = 1.52 × 10^− 5^), ASD (*p*_combined_ = 8.76 × 10^− 5^), and MDD (*p*_combined_ = 4.25 × 10^− 6^). The ARVC pathway describes several processes that are related to heart failure, arrhythmia, and sudden death. Although the pathway itself did not show a clear relationship with neurological/neuropsychiatric traits, several genes in this pathway are frequently observed to be associated with neuronal and brain functions, such as calcium voltage-gated channel subunit genes (*CACNA1C*, *CACNA1D*), catenin genes (*CTNNA1*, *CTNNA2*) and *SLC8A1*. The ARVC pathway has also been previously observed in SCZ [19] and MDD [25]. Among the immune-related traits, the KEGG pathway “JAK-STAT signaling pathway” was ranked the most significant in CD (*p*_combined_ = 2.76 × 10^− 7^), RA (*p*_combined_ = 1.62 × 10^− 5^), and UC (*p*_combined_ = 2.48 × 10^− 9^). This pathway has been previously reported to be involved in the adaptive and mucosal immunity as well as epithelial repair and regeneration [26–28] and is biologically relevant with those immune-related traits. Among the metabolic traits, the Reactome pathway “HDL mediated lipid transport” was found as the most significant pathway in HDL (*p*_combined_ = 2.93 × 10^− 6^) and TC (*p*_combined_ = 7.72 × 10^− 5^), while another much related pathway from Reactome, “chylomicron mediated lipid transport”, was mostly associated with TG (*p*_combined_ = 1.31 × 10^− 6^).

### Genes in the most significantly associated pathways

We next examined the individual genes in each of the most significantly associated pathways. We chose those genes that had a gene-based *p*-value as calculated by PASCAL (denoted by *p*_gene_) less than 1 × 10^− 4^. For the most significantly associated pathways in each of the 25 traits, an average of 13 genes had *p*_gene_ < 1 × 10^− 4^, ranged between 2 (SCZ and LDL) to 69 (HEIGHT). The ratio of genes with *p*_gene_ < 1 × 10^− 4^ in each pathway ranged between 3.8% (AAM) and 60% (HDL), with an average 19% (Additional file 2: Table S2). For example, in the KEGG pathway “folate biosysthesis”, which was the most significantly associated pathway with SCZ (*p*_PASCAL_ = 9.99 × 10^− 4^, *p*_INRICH_ = 4.30 × 10^− 2^, *p*_combined_ = 4.80 × 10^− 4^), there were only two genes that were significantly associated with the trait at the gene level: *SPR* (*p*_gene_ = 5.02 × 10^− 5^) and *GGH* (*p*_gene_ = 8.06 × 10^− 5^). In contrast, in the Reactome pathway “HDL mediated lipid transport”, which was most significantly associated with HDL (*p*_PASCAL_ = 3.39 × 10^− 7^, *p*_INRICH_ = 0.52, *p*_combined_ = 2.93 × 10^− 6^), nine out of 15 genes (60%) in this pathway showed gene-based significance (*p*_gene_ < 1 × 10^− 4^).

**Fig. 4:**
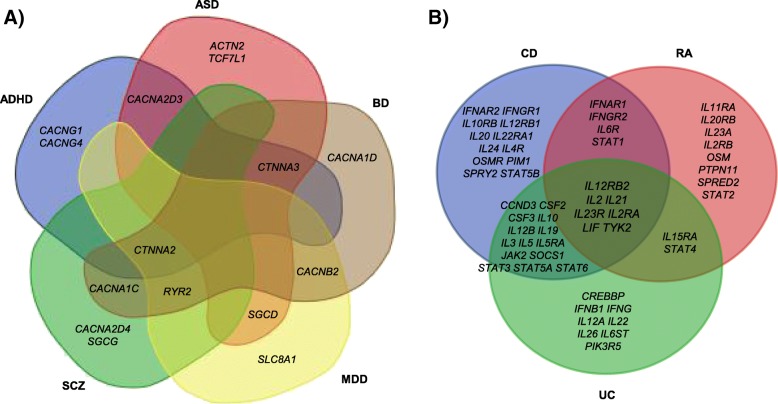
Venn diagram to compare genes in the most significantly associated pathway. **a** Trait-associated genes (*p*_gene_ < 1 × 10^− 4^) from five neuropsychiatric traits in the ARVC pathway. **b** Trait-associated genes from three immune-related traits in the JAK-STAT signaling pathway

One of the advantages of pathway-based analysis is that the combined effect of multiple genes, instead of the effects of each individual gene, was examined for disease association. We thus asked whether the same genes contributed to the disease association at the pathway level. For the neurological/neuropsychiatric traits, we chose the ARVC pathway as an example since this pathway was most significantly associated with 3 out of the 6 traits (the combined *p*-value: *p*_ALZ_ = 4.35 × 10^− 2^, *p*_ADHD_ = 1.52 × 10^− 5^, *p*_ASD_ = 8.76 × 10^− 5^, *p*_BD_ = 6.44 × 10^− 4^, *p*_MDD_ = 4.25 × 10^− 6^, *p*_SCZ_ = 7.49 × 10^− 4^). There were a total of 76 genes in this pathway. As shown in Fig. 4a, a total of 15 genes had gene-based significance (*p*_gene_ < 1 × 10^− 4^) in at least one neuropsychiatric trait. Seven genes were significant in two traits. Each trait has one or two private genes that were uniquely associated with the trait itself but not with other traits. Similarly, we chose the JAK-STAT signaling pathway to examine shared genes for the three immune-related traits (Fig. 4b). There were 155 genes in this pathway, in which 55 genes were associated with at least one trait at *p*_gene_ < 1 × 10^− 4^. We checked the genes that were associated with multiple traits as well as the genes that were uniquely associated with one single trait. Notably, seven genes from this pathway were found to be associated with all three traits: *IL12RB2* (location in the human genome: 1p31.3), *IL2* (4q27), *IL21* (4q27), *IL23R* (1p31.3), *IL2RA* (10p15.1), *LIF* (22q12.2), and *TYK2* (19p13.2). Among them, *IL12RB2* was previously reported to be associated with an autoimmune disease Systemic Lupus Erythematosus in Chinese population [29]; *IL21* plays key roles in humoral immunity and autoimmune diseases [30]; *IL23R* is located in a genomic region that is associated with RA [31]; and *TYK2* had been considered as a potential drug target for certain common autoimmune disorders [32]. However, these seven genes only accounted for 13% of all (55) associated genes in the “JAK-STAT signaling pathway”. Another 14 genes showed gene-based significance across two intestinal diseases (CD, and UC), but not RA. Nevertheless, there were genes private to each of the three traits that were contributive to the pathway, implying heterogamous genetic risks at the gene level which converged at the pathway level. By exploring the disease-associated pathways in all the 25 traits, we found that in most cases, the enrichment of pathway was due to the combined effect of multiple genes, rather than being driven by single genes.

### Cross-trait association at the pathway level

We next explored how multiple traits were related at the pathway level. We first conducted a preliminary screen using the full-spectrum of *p*-values for all pathways and the hierarchical clustering analysis. As shown in Additional file 3: Figure S1, we observed an obvious trend that biologically relevant traits tended to cluster together. To accurately estimate the functional overlapping of genetic variants at the pathway level, we next exploited FET for all possible pairs of traits. Results from FET were summarized in Additional file 4: Table S3, including the lists of shared pathways and all statistics. The magnitude of overlapping pathways varied greatly. At adjusted *p*_FET_ < 1 × 10^− 3^, the number of shared significant pathways (*p*_combined_ < 0.01) between any pair of traits ranged between 12 (FG and FI) to 99 (UC and CD). These shared pathways proved that trait-associated genetic variants indeed converged on functional pathways that potentially contributed to the traits. Because these pathways were nearly non-redundant (i.e., with < 30% overlapping genes per pair), they represent assessment of independent functional processes.

**Fig. 5:**
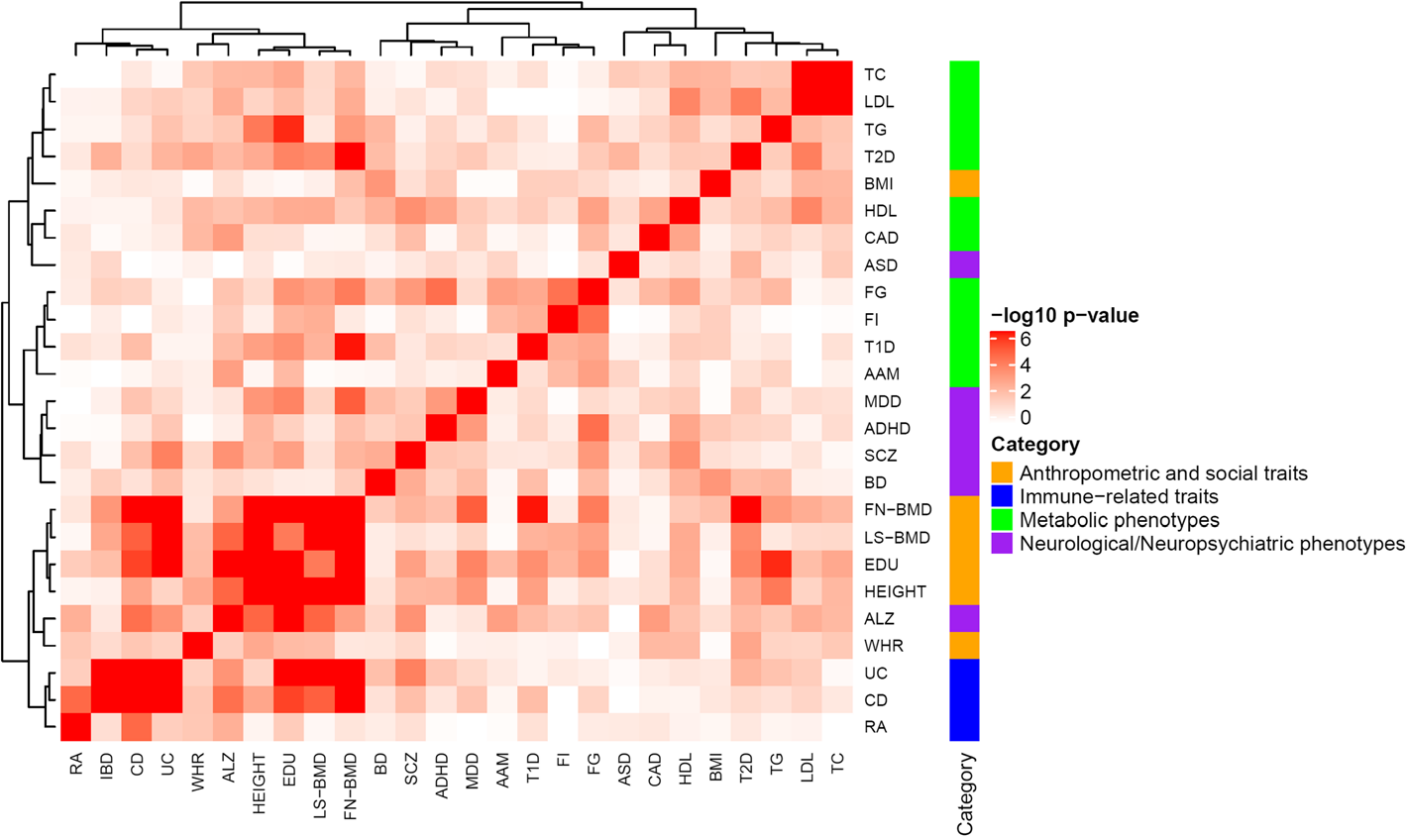
Heatmap of 25 traits. Hierarchical clustering was performed using *p*-values from Fisher’s Exact Test

Using hierarchical clustering, the 25 traits could be visually distinguishable through forming sub-groups that were quite consistent with their pre-assigned group labels (Fig. 5). The immune-related traits all clustered with each other and they are closest to the anthropometric and social group. The anthropometric and social traits also formed a sub-group, except BMI that was distantly located. The immune group and the neurological/neuropsychiatric group were close to each other, which was expected because many previous works have reported that immune related processes are widely involved in several neurological/neuropsychiatric traits such as SCZ [33] and ALZ [34].

To further explore which pathways contributed to the trait associations, we next examined the 20 most significantly associated pathways within each group of traits (ordered by the average *p*_combined_ across traits within group, Additional file 5: Figure S2). In the neurological/neuropsychiatric trait group, we found several pathways that were shared by multiple traits and were also previously implicated. For example, the KEGG “calcium signaling pathway” (*p*_ALZ_ = 5.59 × 10^− 5^, *p*_ADHD_ = 2.96 × 10^− 4^, *p*_ASD_ = 1.88 × 10^− 1^, *p*_BD_ = 8.61 × 10^− 5^, *p*_MDD_ = 6.20 × 10^− 5^, *p*_SCZ_ = 1.62 × 10^− 3^), the KEGG “axon guidance” pathway (*p*_ALZ_ = 1.07 × 10^− 3^, *p*_ADHD_ = 8.12 × 10^− 4^, *p*_ASD_ = 6.22 × 10^− 4^, *p*_BD_ = 1.13 × 10^− 2^, *p*_MDD_ = 2.62 × 10^− 3^, *p*_SCZ_ = 2.05 × 10^− 3^), the “alanine aspartate and glutamate metabolism” (*p*_ALZ_ = 9.60 × 10^− 3^, *p*_ADHD_ = 8.22 × 10^− 2^, *p*_ASD_ = 1.22 × 10^− 2^, *p*_BD_ = 5.31 × 10^− 3^, *p*_MDD_ = 6.93 × 10^− 3^, *p*_SCZ_ = 1.70 × 10^− 2^), and “the voltage gated potassium channels” (*p*_ALZ_ = 2.04 × 10^− 3^, *p*_ADHD_ = 2.33 × 10^− 4^, *p*_ASD_ = 3.16 × 10^− 4^, *p*_BD_ = 2.86 × 10^− 2^, *p*_MDD_ = 4.69 × 10^− 3^, *p*_SCZ_ = 5.80 × 10^− 3^) were nominally significantly associated with multiple traits and were all previously implied in neuropsychiatric disorders [19, 35]. In the metabolism group, we observed pathways related to lipid metabolism and were shared by all four traits: “chylomicron mediated lipid transport” (*p*_HDL_ = 3.54 × 10^− 4^, *p*_LDL_ = 1.09 × 10^− 3^, *p*_TC_ = 1.09 × 10^− 4^, *p*_TG_ = 1.32 × 10^− 6^), “HDL mediated lipid transport” (*p*_HDL_ = 2.93 × 10^− 4^, *p*_LDL_ = 1.97 × 10^− 3^, *p*_TC_ = 7.72 × 10^− 4^, *p*_TG_ = 2.71 × 10^− 6^), “alpha Q signaling events” (*p*_HDL_ = 2.35 × 10^− 3^, *p*_LDL_ = 3.16 × 10^− 3^, *p*_TC_ = 3.12 × 10^− 3^, *p*_TG_ = 1.75 × 10^− 3^), and “ubiquitin mediated proteolysis” (*p*_HDL_ = 1.96 × 10^− 4^, *p*_LDL_ = 8.92 × 10^− 4^, *p*_TC_ = 9.90 × 10^− 4^, *p*_TG_ = 2.75 × 10^− 3^).

**Fig. 6:**
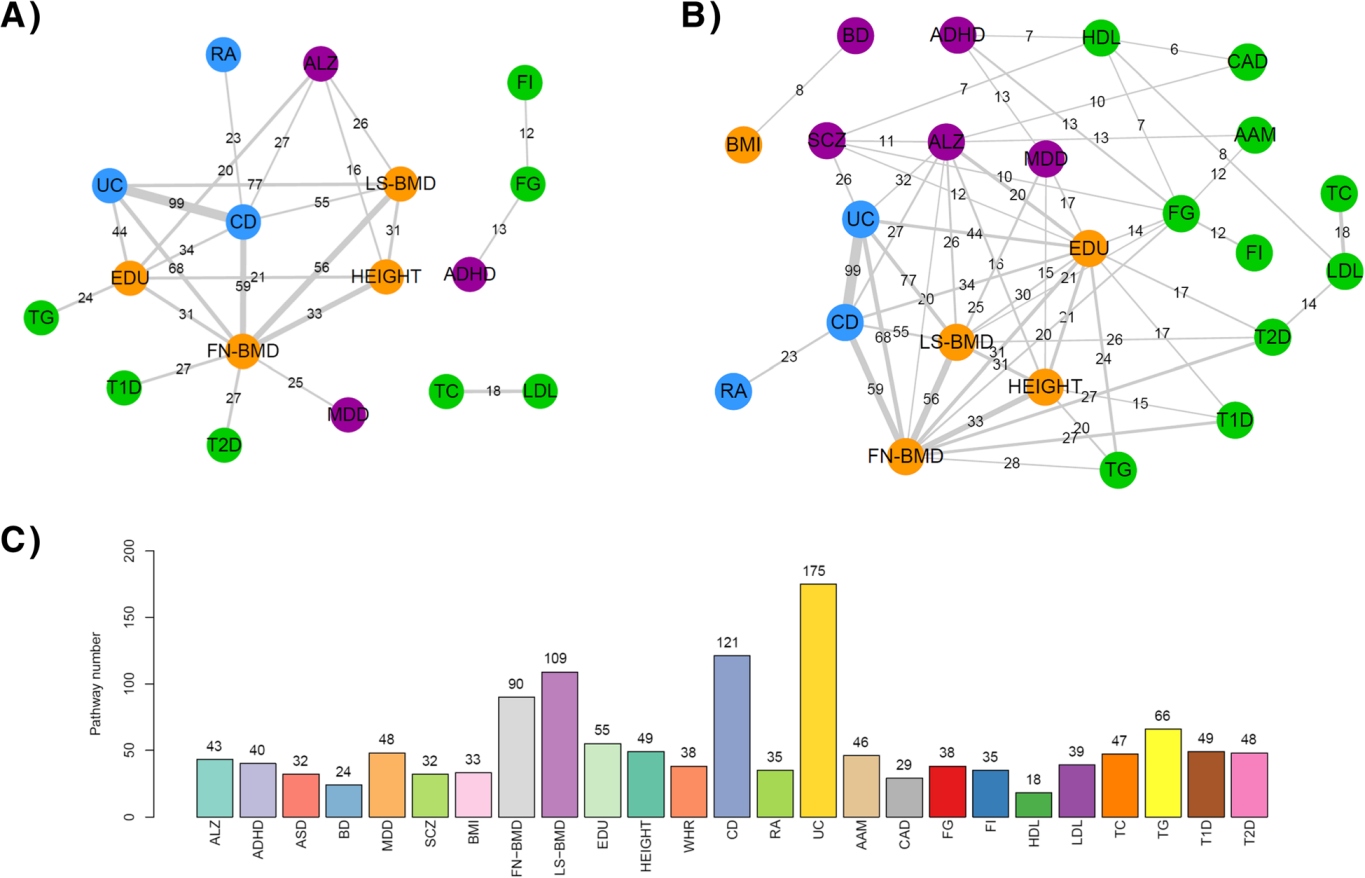
Trait-trait association network based on Fisher’s Exact Test. **a** Network obtained using adjusted *p*_FET_ < 1 × 10^− 3^. **b** Network obtained using adjusted *p*_FET_ < 0.01. Each node represents a trait. Node color indicates trait group (purple: neurological/neuropsychiatric traits, orange: anthropometric and social traits, blue: immune-related traits, and green: metabolic traits). Edge indicates the two end traits shared a significant number of pathways. Edge width is proportional to the -log_10_-transformed adjusted *p*_FET_. Edge labels represent the number of shared associated pathways. (**C**) The number of pathways significantly associated with each trait (*p*_combined_ < 0.01)

To better represent the relationship among the multi-traits, we built a trait-trait association network based on the FET result using two thresholds: adjusted *p*_FET_ < 1 × 10^− 3^ (referred as highly significantly connected network) and adjusted *p*_FET_ < 0.01 (referred as moderately significantly connected network). After applying the adjusted *p*_FET_ < 1 × 10^− 3^ (Fig. 6a), we observed strong associations (i.e., edges) among pairs of immune-related traits and anthropometric and social traits. The immune related traits in general had more associated pathways individually and more shared pathways across traits (Fig. 6c). UC and CD as the two most common form of IBD shared 99 pathways (adjusted *p*_FET_ = 8.30 × 10^− 17^). In addition, we observed crosstalk between bone mineral density (LS-BMD and FN-BMD) and immune-related traits (UC and CD) [36]. FN-BMD shared 25 to 68 pathways with traits from other groups (i.e., UC, CD, T1D, T2D and MDD). This observation is consistent with increased likelihood of osteoporosis among those with type 1 diabetes from the civilian community in the United States [37]. We observed 25 shared pathways between FN-BMD and MDD. This strong sharing, although unexpected, had some support from previous work that bone mineral density was reduced in patients with depressive disorders [38]. For other closely related traits, we found LDL and TC shared 18 pathways (adjusted *p*_FET_ = 2.29 × 10^− 6^) and FG and FI shared 12 pathways (adjusted *p*_FET_ = 7.36 × 10^− 4^). Interestingly, at adjusted *p*_FET_ < 1 × 10^− 3^, we did not observe any significant association among neurological/neuropsychiatric traits, although association between ALZ, ADHD, and MDD with traits of other groups were observed (e.g., MDD versus FN-BMD, ADHD and FG).

When we used a relaxed threshold (adjusted *p*_FET_ < 0.01), we observed more associations (Fig. 6b). In addition to the immune-related group and the social related group, traits from the metabolic group and traits from the neurological/neuropsychiatric group were each found to have strong within-group connections. For example, SCZ shared 11 pathways with ALZ (adjusted *p*_FET_ = 3.89 × 10^− 3^) and MDD and ADHD shared 13 pathways (adjusted *p*_FE_ = 5.61 × 10^− 3^). Interestingly, SCZ and UC shared 25 associated pathways (adjusted *p*_FET_ = 1.52 × 10^− 3^). This observation is consistent with increased rate of autoimmune diseases in schizophrenia patients in Denmark [39, 40] and with molecular evidence for a partial autoimmune etiology for schizophrenia [41]. Finally, BD and BMI shared 8 common associated pathways (adjusted *p*_FET_ = 4.59 × 10^− 3^), where BD patients were found with higher BMI and increased metabolic comorbidity in previous studies [42]. ALZ and CAD had 10 common associated pathways (adjusted *p*_FET_ = 5.94 × 10^− 3^), consistent with the previous finding that many cardiovascular risk factors are shown to increase the risk of dementia and ALZ [43]. Figure 6c illustrated the total number of associated pathways for each trait**.**

## Discussion

During the past decade, large-scale GWAS data for complex diseases and traits have been growing at an unprecedented rate [44]. While conventional GWAS analyses apply genome-wide significance thresholds (e.g., 5 × 10^− 8^) to select genetic variants for further investigation [45], pathway-based analysis presents a complementary way to explore GWAS data by examining groups of functional related genes for their combined effects, and thus, it has the improved power and interpretability [14, 18, 19, 25, 35, 46]. In this study, we take the advantage of pathway-based analysis to study cross-trait associations. We developed a pipeline to integrate pathway enrichment results from two independent methods using large-scale GWAS summary statistics for 25 traits. The two methods differ in their assumptions and individual strengths and thus, provided complementary assessment of trait-associated pathways. Further boosted by the Fisher’s combined probability test and trait-trait association network, we examined shared pathways, contributing genes, and cross-trait associations. Our results provided insights into the functional association based on genetic variants and can be extended when more GWAS summary data are made available in future.

Our primary goal was to use individual variants found to be associated with multiple traits to identify shared biological pathways between traits, as we hypothesized that similar or related pathways are likely underlie the pathogenicity of related traits. In our results, we found the immune-related traits shared many trait-associated pathways. This finding was consistent with previous studies that immune-related traits tended to have many genetic markers with common function in immunity and inflammation [3]. In addition, we observed a number of highly relevant relationships between multiple complex traits by associated pathway enrichment results, while most genes in the pathway did not show significant overlap. For example, the anthropometric trait, FN-BMD, was found to share pathways with traits from other groups such as T1D [47], T2D [48], MDD [38] and UC [36]. Although such associations have been reported before, the underlying mechanisms remain unknown. Through our analyses, we not only presented further evidence at the pathway level to these associations, but also revealed biological pathways in which the genetic variants converged. For example, FN-BMD shared 27 pathways with T1D and 27 pathways with T2D respectively; among them, 9 pathways were shared among all three traits. Overall, we demonstrated that biologically relevant traits had stronger sharing at the pathway level than at the gene level and our pathway results would provide further interpretation of genetic variants that drive the cross-trait associations.

Our work has several limitations. First, the current pathway resources that are publically available and widely used in the field such as KEGG unavoidably have limitations such as low resolution in defining biological pathways, relatively few genes compared to genome-wide datasets, overrepresentation of well-known pathways versus underrepresentation of pathways attracting less common interests, and lack of tissue and cell specificity, among others. All our analyses were confined with these limitations. Second, we used 3 pathway databases to increase the use of annotated pathways. However, many pathways may be redundant when more pathways are included. We evaluated and removed those highly redundant pathways (30% gene). This may exclude lots informative pathways in our analyses. However, Fisher’s Exact Test used in this study still with limitation as all associated pathway are not completely independent. Therefore, it is still a difficult task to balance the pathway independence and information. Third, pathway-based analysis can only evaluate genetic variants located in gene regions but ignore the majority of variants located in the non-coding regions. As a result, there is information lost for variants located at non-coding regions or genes without any pathway information. Fourth, our work focused on common variants based on genotyping arrays and imputation, with MAF > 0.05. Inferences from rare variants may also provide insights into cross-trait association but were not investigated in our work (e.g., for traits under strong negative selection [5, 49]). Despite these challenges, our work provides a complementary way to utilize GWAS data to study cross-trait associations at the pathway level, which is a promising strategy to discover novel biological insights underlying complex traits.

## Conclusions

We presented an integrative framework to systematically investigate cross-trait associations using GWAS summary statistics for 25 traits using pathway-based analysis. Our results revealed trait-associated pathways individually and in common, highlighting associations both within and across trait groups. We observed a significant proportion of pathways shared by the immune-related traits and anthropometric and social traits, and a number of moderately significant correlations within neurological/neuropsychiatric disorder and metabolic phenotypes. Interestingly, more than half of the associations were cross trait groups. We also constructed a novel trait-trait association network to better examine the genetic association at the pathway level. These results collectively shed lights on the molecular pathways underlying complex traits, both related or unrelated traits. The results warrant further investigation of function of critical nodes (genes) or validated when more data become available.

## Methods

### Data collection

GWAS summary statistics were downloaded for 25 different traits from International Genomics of Alzheimer’s Project (IGAP, http://web.pasteur-lille.fr/en/recherche/u744/igap/igap_download.php), Genetic Factors of Osteoporosis (GEFOS, http://www.gefos.org) consortium, The Genetic Investigation of ANthropometric Traits (GIANT, https://portals.broadinstitute.org/collaboration/giant/index.php/Main_Page), International Inflammatory Bowel Disease Genetics Consortium (IIBDGC, https://www.ibdgenetics.org/downloads.html), Meta-Analyses of Glucose and Insulin-related traits Consortium (MAGIC, https://www.magicinvestigators.org), Psychiatric Genomics Consortium (PGC, http://www.med.unc.edu/pgc), Reproductive Genetics Consortium (ReproGen, [50]) and Social Science Genetic Association Consortium (SSGAC, https://www.thessgac.org). These traits span a wide range of phenotype measurements categorized into four groups. We used the samples of European ancestry. For genotyped SNPs, we removed those with minor allele frequency (MAF) < 0.05. Wherever applicable, we mapped GWAS summary data to the hg19 human assembly using *liftOver* software [51].

### Pathway enrichment analysis

In our analysis using PASCAL, we chose SNPs with nominal significance of association (GWAS *p*-value < 0.01). This is a commonly used strategy to find SNPs with weak or moderate association signals before they can be integrated for further analysis at the pathway level [19]. SNPs were mapped to genes if they were located within a range of 50 kb upstream or downstream of genes’ transcription start sites. PASCAL employs a chi-squared method that takes into account of LD, gene length, and SNP density [12]. INRICH [20] considers SNPs that are associated with a trait at *p*-value < 1 × 10^− 4^ (in our case, we used *p*-value < 1 × 10^− 2^) and generates independent genomic intervals for the enrichment test. The Plink clumping function was employed, taking the 1000 Genomes Project European panel as the reference and the following parameters: r^2^ = 0.2, maximum distance between a pair of SNPs is 1 Mb. The aim to generate LD-independent intervals was to ensure that SNPs located adjacent in the human genome are analyzed as one independent unit as they might tag the same causal variants. INRICH overlaps the independent intervals with the pre-defined pathways and calculate an enrichment statistic *E* for each pathway, where *E* is the number of intervals that overlap at least one target gene in the pathway. To estimate the significance of *E*, a permutation test is conducted to generate a null set of intervals which match the interval size, overlapping gene, and SNP density to the original input intervals [52]. We chose to generate 1000 replicates to calculate the empirical *p*-value for each pathway.

With the successful accomplishment of both PASCAL and INRICH, we had two *p*-values for each pathway in each trait, testing the same hypothesis that whether an investigated pathway is associated with the trait of interest. Thus, we introduced the Fisher’s combined probability test to calculate a combined *p*-value for each pathway in each treat as follows: ${\chi}_{2k}^2=-2{\sum}_{i=1}^k\ln \left({p}_i\right)$, where *χ*^2^ follows a chi-squared distribution with 2 *k* degrees. In our case *k* = 2.

### Reduce redundancy among pathway annotations

From an initial compiled set of 1077 pathways across three gene set databases (KEGG, BioCarta, Reactome), we restricted downstream analyses to 1032 pathways of size 10–200 genes. We further removed pathways that showed substantial redundancy, i.e., any two pathways sharing more than 30% genes (# shared genes /max{# genes in pathway A, # genes in pathway B}) were identified and the larger pathway would be retained. Finally, a total of 355 pathways assumed as non-redundant were obtained and used for follow up analysis, including 67 KEGG, 79 BioCarta and 209 Reactome pathways.

### Cross-trait association based on trait-associated pathways

To assess whether a pair of traits share pathways higher than chance expectation, we first determined the pathways that were significantly associated with each trait (*p*_combined_ < 0.01), followed by Fisher’s Exact Test. Specifically, for two traits *a* and *b*, we build a dichotomous 2 × 2 contingency table as follows:

**Table:** 

	Trait *b*	
Trait *a*	∣*A* ∩ *B*∣	$\mid \overline{A}\cap B\mid$
	$\mid A\cap \overline{B}\mid$	$\mid \overline{A}\cap \overline{B}\mid$

Here, *A* denotes the set of pathways significantly associated with trait *a* and *B* denotes the set of pathways significantly associated with trait *b*. The format |•| indicates the number of records referred by •. ∣A ∩ B∣ denotes the number of pathways significantly associated with both traits *a* and *b*, $\mid \overline{A}\cap B\mid$and $\mid A\cap \overline{B}\mid$ denote the number of pathways showing association only in trait *b* or *a*, respectively, and $\mid \overline{A}\cap \overline{B}\mid$ represents the number of pathways that are not associated with either trait. We used Fisher’s Exact Test to estimate whether two traits shared overrepresented pathways.

Hierarchical clustering was then conducted based on the FET results. We used Spearman distance and ward linkage aggregation. Heatmaps were created using the *ComplexHeatmap* package in *R*. In our analysis, we consider a level of significance with adjusted *p* < 0.01 using the Benjamini and Hochberg’s method [22] to correct multiple tests.

## Additional files

Additional file 1: **Table S1.** Results of pathway-based analysis of the 25 traits. (XLSX 152 kb)

Additional file 2: **Table S2.** Significantly associated genes in the most significant pathway for each trait. (XLSX 14 kb)

Additional file 3: **Figure S1.** Hierarchical clustering analysis for all associated pathways between 25 traits. (PDF 85 kb)

Additional file 4: **Table S3.** Results of Fisher’s Exact Test for 25 traits. (XLSX 46 kb)

Additional file 5: **Figure S2.** Heatmap showing the 20 most significant pathways for each trait group. (PDF 539 kb)

## Abbreviations

AAM: Age at menarche
ADHD: Attention deficit-hyperactivity disorder
ALZ: Alzheimer’s disease
ASD: Autism spectrum disorder
BD: Bipolar disorder
BMI: Body mass index
CAD: Coronary artery disease
CD: Crohn’s disease
EDU: Educational attainment
FDR: False discovery rate
FET: Fisher’s Exact Test
FG: Fasting glucose
FI: Fasting insulin
FN-BMD: Bone mineral density (femoral neck)
GWAS: Genome-wide association study
HDL: High-density lipoproteins
IBD: Inflammatory bowel disease
LD: linkage disequilibrium
LDL: Low-density lipoproteins
LS-BMD: Bone mineral density (lumbar spine)
MAF: Minor allele frequency
MDD: Major depressive disorder
MDS: Multi-dimensional scaling
RA: Rheumatoid arthritis
SCZ: Schizophrenia
SNP: Single nucleotide polymorphism
T1D: Type 1 diabetes
T2D: Type 2 diabetes
TC: Total cholesterol
TG: Triglycerides
UC: Ulcerative colitis
WHR: Waist–hip ratio
