# Supplementary figures and images for: Investigation of multi-trait associations using pathway-based analysis of GWAS summary statistics
Source: BMC Genomics. 2019 Feb 4;20(Suppl 1):79. doi: 10.1186/s12864-018-5373-7 (PMC6360716; doi:10.1186/s12864-018-5373-7)

Additional file 3. Figure S1

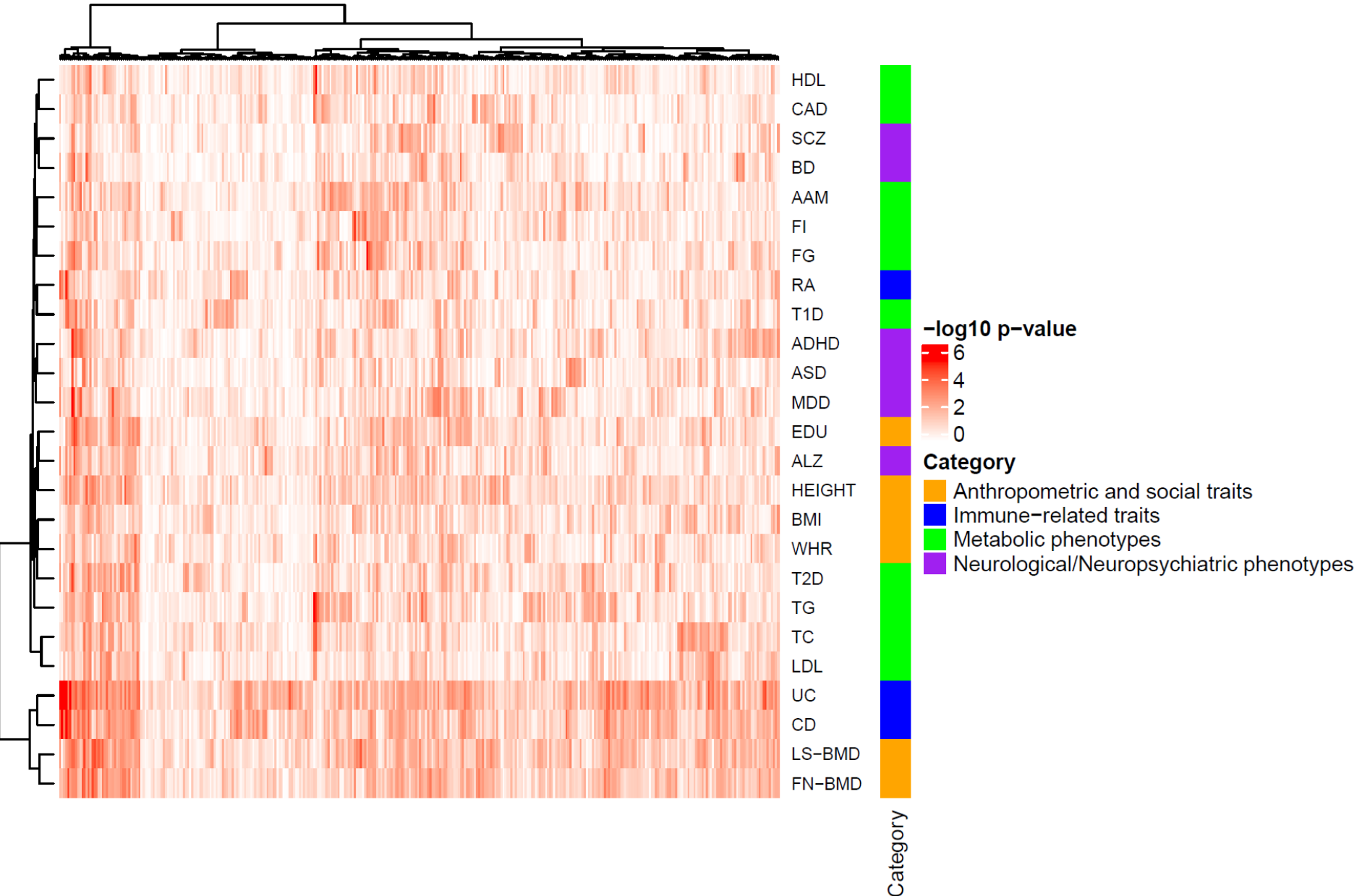

Supplement: Supplementary file 3 — Figure S1. Hierarchical clustering analysis for all associated pathways between 25 traits. (PDF 85 kb) [file 12864_2018_5373_MOESM3_ESM.pdf]

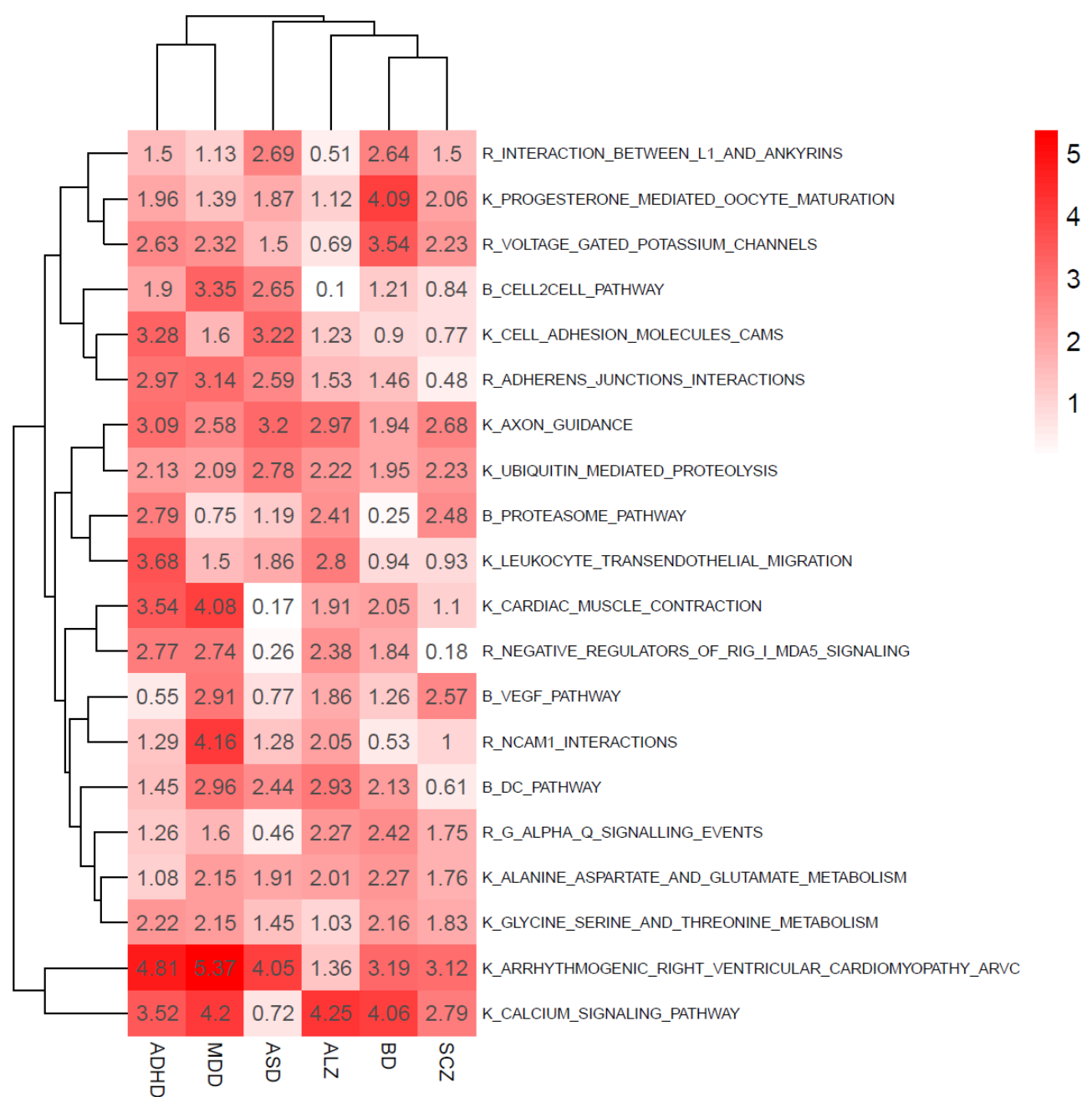

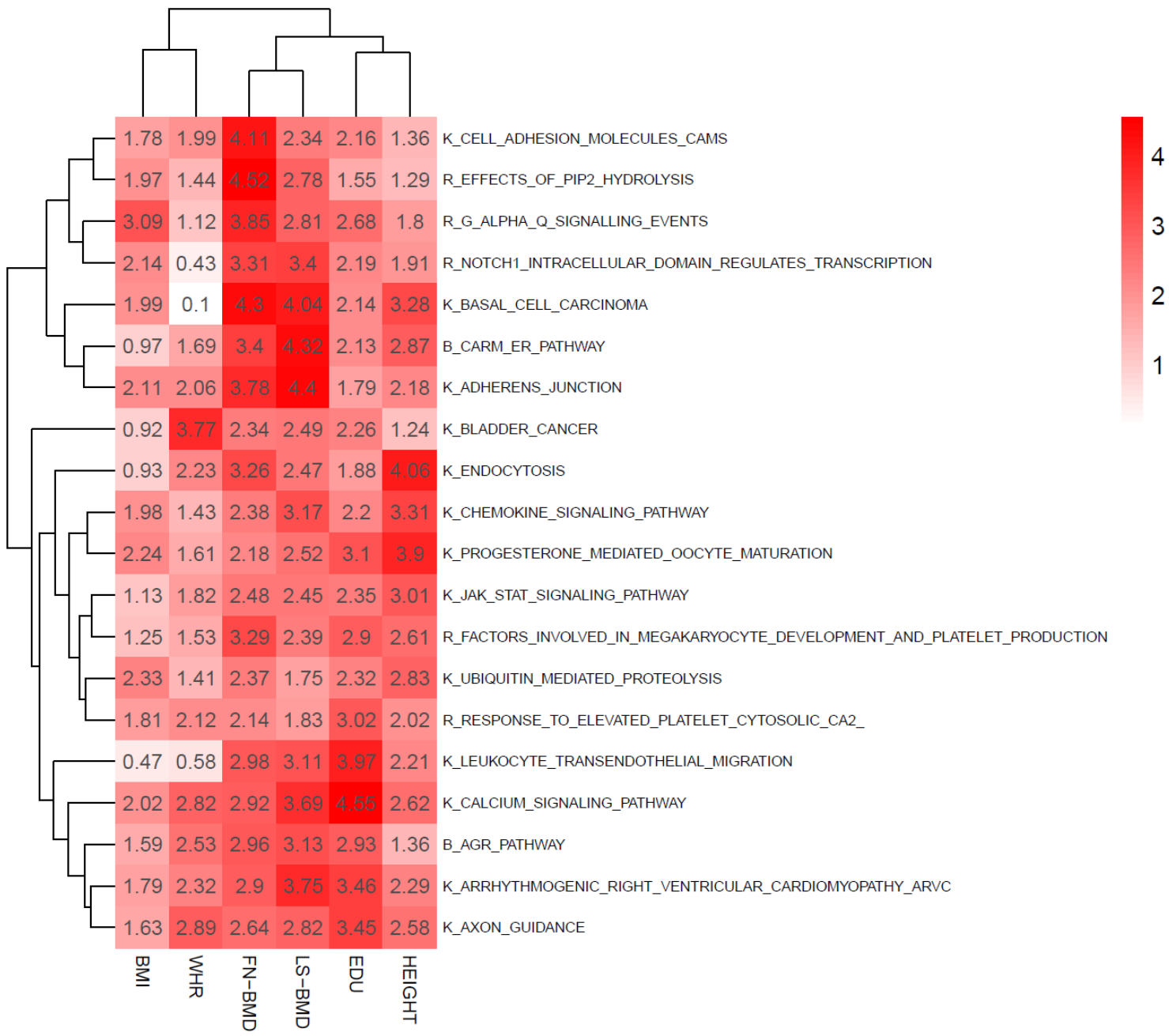

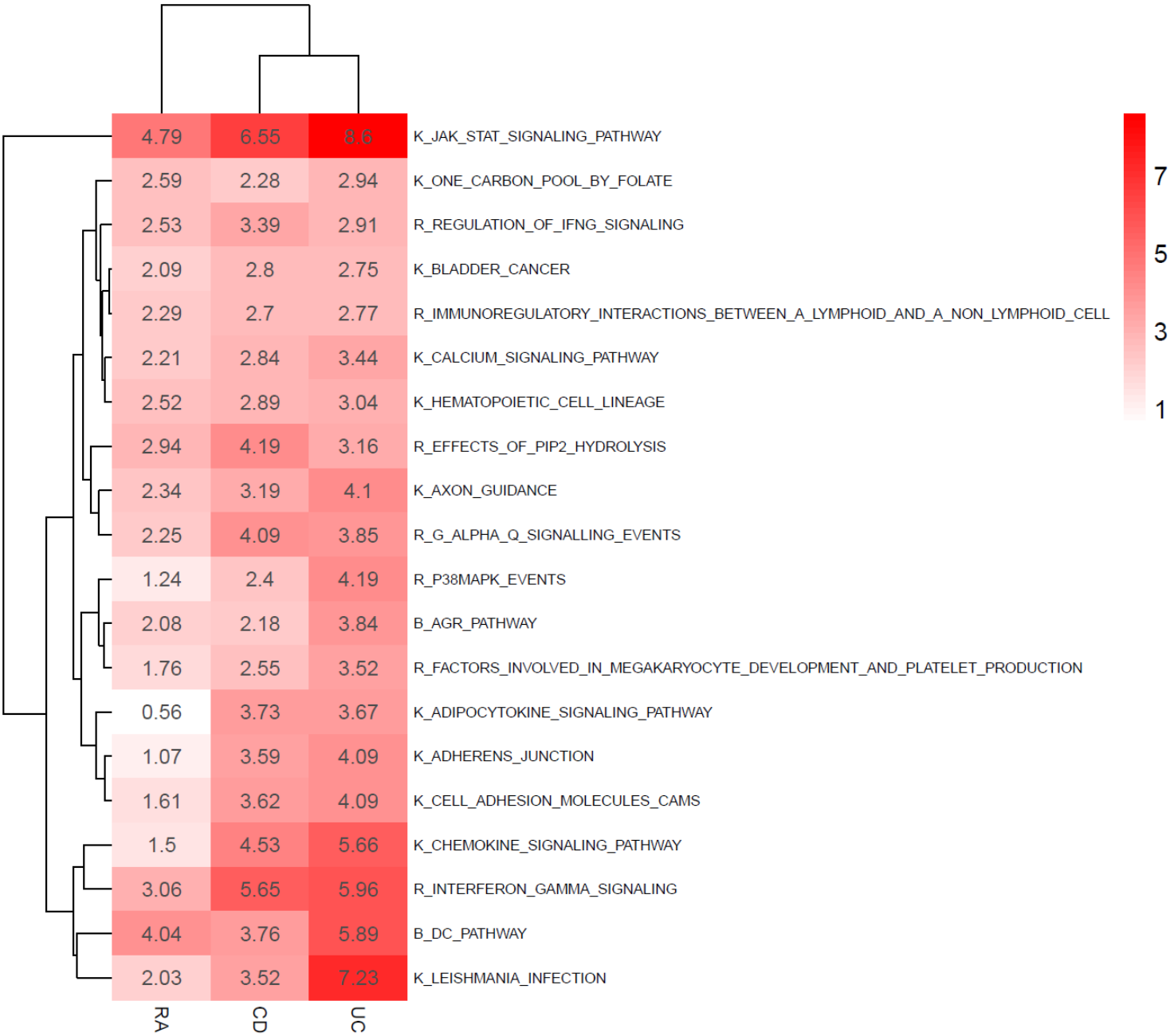

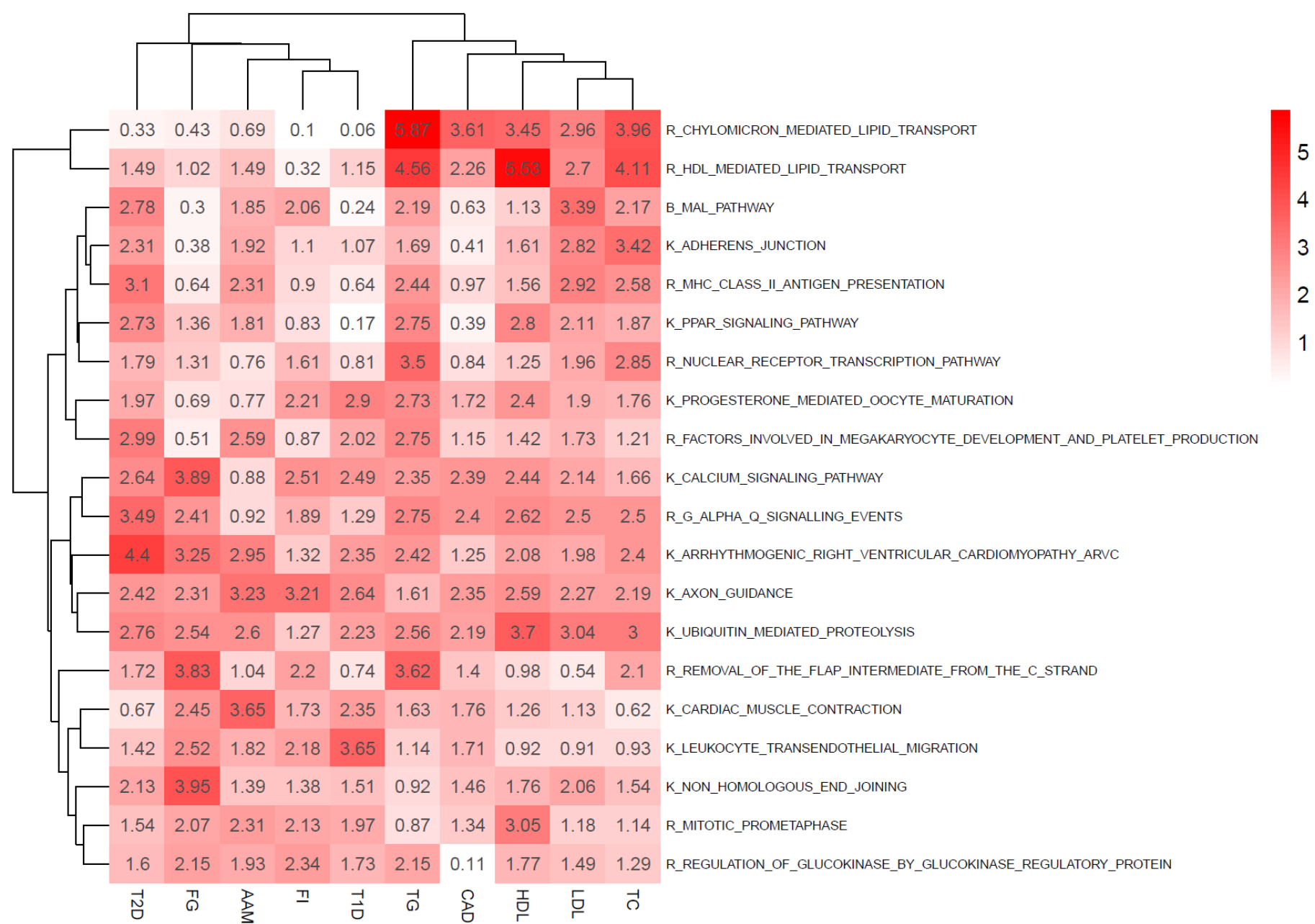

Supplement: Supplementary file 5 — Figure S2. Heatmap showing the 20 most significant pathways for each trait group. (PDF 539 kb) [file 12864_2018_5373_MOESM5_ESM.pdf]
